# Nest destruction elicits indiscriminate con- versus heterospecific brood parasitism in a captive bird

**DOI:** 10.1002/ece3.1243

**Published:** 2014-11-19

**Authors:** Rachael C Shaw, William E Feeney, Mark E Hauber

**Affiliations:** 1 School of Biological Sciences, University of AucklandAuckland, New Zealand; 2 School of Biological Sciences, Victoria University of WellingtonWellington, New Zealand; 3 Research School of Biology, Australian National UniversityCanberra, Australia; 4 Evolutionary Ecology Group, Department of Zoology, University of CambridgeCB23EJ, United Kingdom; 5 Department of Psychology, Hunter College and the Graduate Center, City University of New YorkNew York, USA

**Keywords:** Brood parasitism, estrildid, intraspecific brood parasitism, interspecific brood parasitism, nest predation

## Abstract

Following nest destruction, the laying of physiologically committed eggs (eggs that are ovulated, yolked, and making their way through the oviduct) in the nests of other birds is considered a viable pathway for the evolution of obligate interspecific brood parasitism. While intraspecific brood parasitism in response to nest predation has been experimentally demonstrated, this pathway has yet to be evaluated in an interspecific context. We studied patterns of egg laying following experimental nest destruction in captive zebra finches, *Taeniopygia guttata*, a frequent intraspecific brood parasite. We found that zebra finches laid physiologically committed eggs indiscriminately between nests containing conspecific eggs and nests containing heterospecific eggs (of Bengalese finches, *Lonchura striata* vars. *domestica*), despite the con- and heterospecific eggs differing in both size and coloration. This is the first experimental evidence that nest destruction may provide a pathway for the evolution of interspecific brood parasitism in birds.

## Introduction

The evolutionary pathways from obligate parental care to obligate interspecific brood parasitism in birds are controversial and enigmatic. Some theories consider interspecific brood parasitism to have evolved directly among parental, nonparasitic ancestors (Yom-Tov and Geffen 2006), while others suggest that intraspecific parasitism can act as an evolutionary “stepping-stone” to obligate parasitism (Hamilton and Orians 1965; Lyon and Eadie 1991; Robert and Sorci 2001). Regardless, facultative brood parasitism appears to be important in the evolution of interspecific parasitism (Cichoń 1996).

Facultative brood parasitism can be a response to unpredictable ecological conditions (Hamilton and Orians 1965; Nolan and Thompson 1975; Sorenson 1991, 1993). For example, Hamilton and Orians (1965) suggested that the laying of physiologically committed eggs in the nests of other birds following nest predation may be a viable pathway to the evolution of intraspecific and obligate interspecific brood parasitism (“Hamilton–Orians” hypothesis). This evolutionary hypothesis has strong support from theoretical modeling studies (Cichoń 1996; Robert and Sorci 2001) and has been demonstrated experimentally in an intraspecific parasitism context: captive zebra finches (*Taeniopygia guttata*) preferentially parasitized the active nests of conspecifics, as opposed to empty nests, following the removal of their own nest during the egg laying period (Shaw and Hauber 2009, 2012). In the field, both starlings (*Sturnus vulgaris*) and moorhens (*Gallinula chloropus*) responded to the experimental removal of partially completed clutches by laying eggs in the active neighboring nests of conspecifics (Feare 1991; McRae 1998). However, an attempt to experimentally evaluate whether nest predation induced parasitic laying in red-winged blackbirds (*Agelaius phoeniceus*) in an intraspecific context in the field was unsuccessful (Rothstein 1993), possibly because this species does not regularly engage in facultative intraspecific brood parasitism (Rothstein 1994; Yezerinac and Dufour 1994). However, the role of nest predation in promoting brood parasitism has yet to be investigated in an interspecific context.

**Figure 1:**
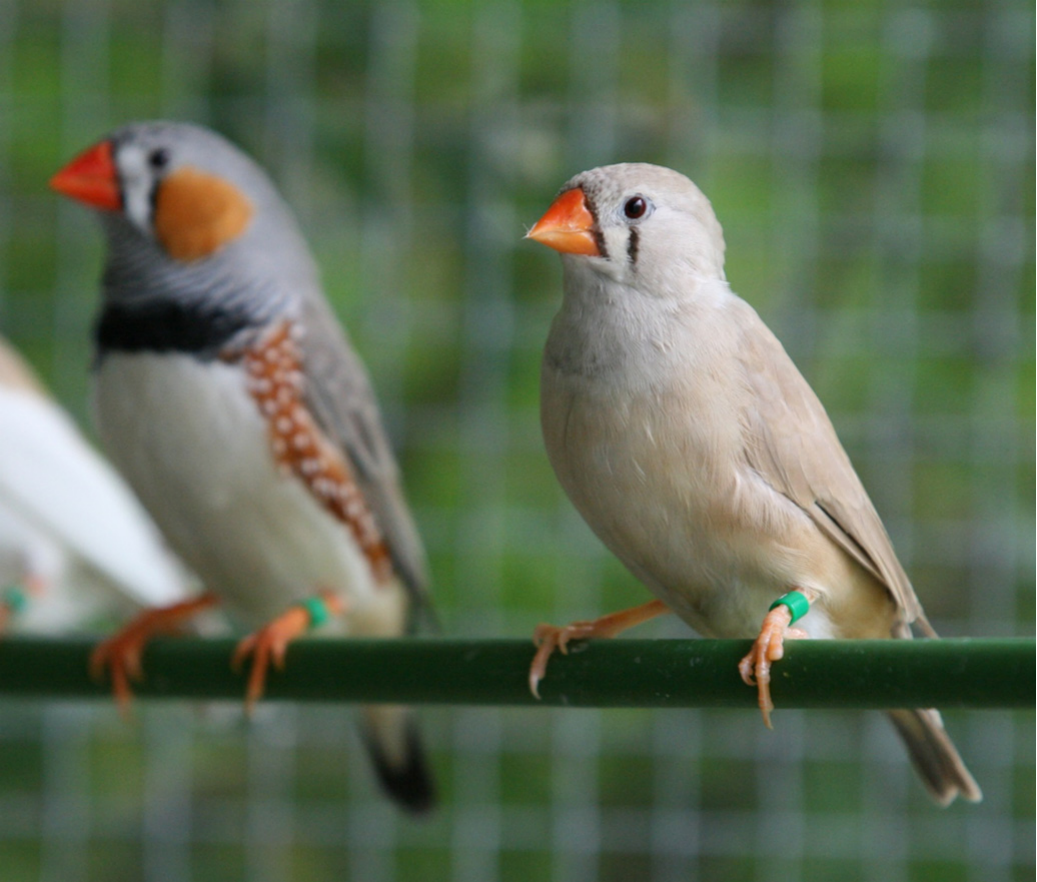
A female zebra finch with a wild type male finch visible in the background (Photo credit: Dana Campbell).

Using artificial nest predation experiments, here we tested whether captive zebra finches (Fig.1), a known facultative intraspecific brood parasite both in the wild (Birkhead et al. 1990; Griffith et al. 2010) and in captivity (Schielzeth and Bolund 2010), would preferentially parasitize a nest containing conspecific eggs or a nest containing heterospecific eggs (from a related estrildid species, Bengalese finch, *Lonchura striata* vars. *domestica*). If zebra finches can recognize egg phenotypes and base a parasitism decision on the perceptual distance between the potential host's egg and their own egg's phenotype, or on generally desirable egg attributes (e.g., larger egg size, Tinbergen 1951; or color, Honza et al. 2014), we would expect preferential parasitism of either the nest containing conspecific eggs or the nest containing heterospecific eggs. Alternatively, incipient parasites may not discriminate between nests containing con- or heterospecific eggs. Indiscriminate laying could occur if facultative parasitism following nest destruction is simply a response to the need to lay physiologically committed eggs into active nests that may contain eggs. Such indiscriminate laying could predispose a species to engage in facultative and, eventually, obligate interspecific brood parasitism.

## Materials and Methods

We conducted experiments between June and October 2008 at the University of Auckland, New Zealand. Birds were color-banded and pairs (*N*  = 17) were housed in individual cages (35 × 30 × 45 cm) that were visually, but not acoustically, isolated from one another (see Shaw and Hauber 2009 for further husbandry details). All birds had previously paired, built nests and laid eggs while they participated in other experiments assessing the prevalence of intraspecific brood parasitic behaviors (Shaw and Hauber 2009, 2012). All protocols were approved by the University of Auckland Animal Ethics Committee.

**Figure 2:**
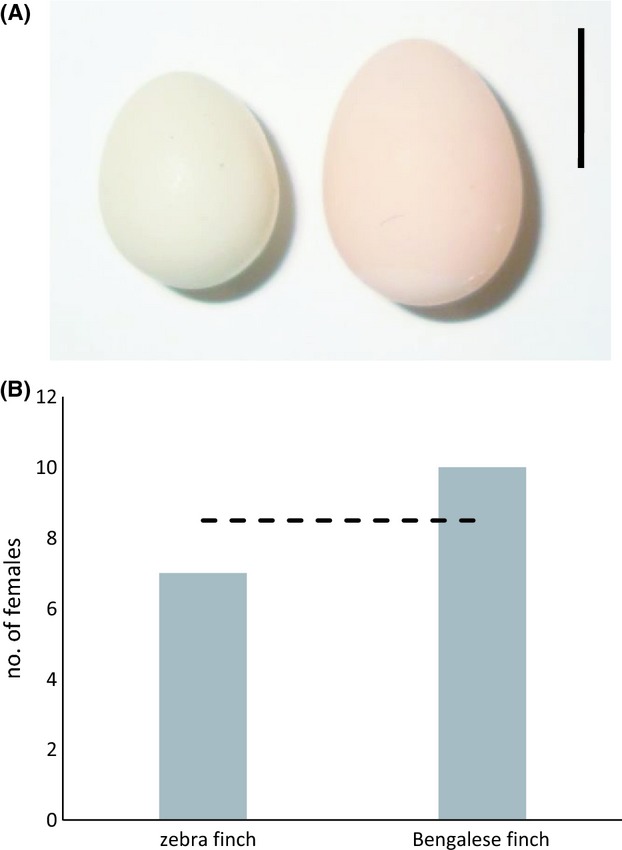
(A) A zebra finch egg (left) and a Bengalese finch egg (right). The scale bar shows 1 cm. (B) The number of host nests into which females laid physiologically committed eggs. The dashed line represents the expected nest choices under the random expectation (50%).

At the start of the experiment, we added three identical artificial enclosed nests (12 × 9 × 9 cm) lined with hay and wool (weight: 10 g, 60% hay, 40% wool) to each pair's cage. We supplied additional lining material *ad libitum* inside the cage until the female began laying in one nest. For each egg laid by the female in her own nest, on the same day, we added one Bengalese finch egg to a nest randomly chosen out of the two remaining nests in the cage and one zebra finch egg to the other. Dummy eggs (Fig.2A) were collected from pairs not participating in the experiment and from a Bengalese finch breeder (D. Campbell) and were refrigerated until used.

We removed the pair's own, active nest on the evening of the second day of the female laying (i.e., when all three nests in the pair's cage each contained two eggs), 2–3 h. before the lights were scheduled to be turned off. The following morning we visually inspected the two remaining nests in the cage 2 h after the lights were turned on and every hour thereafter until the female laid an egg. Each pair yielded only a single data point. After the manipulation, nests were left in the cage for several days to allow females to complete the laying of a clutch (females laid an additional mean 2.3 ± 0.2 SE eggs).

Some birds find larger eggs desirable (Tinbergen 1951) and so we measured and compared the volume of 10 fresh, unincubated Bengalese eggs (from three different females) and 10 fresh zebra finch eggs (from five different females). We photographed eggs with a Sony cybershot (Sony Corp., Tokyo, Japan) digital camera and used ImageJ 1.40 g to measure the egg width and length. We then calculated egg volume (*V*) using the formula, *V * = * * 0.51 *L* (*B*)^2^, where *L* is the length, *B* is the width at the widest point, and 0.51 is an experimentally determined constant (Hoyt 1979). Similarly, as the degree of color matching between host and parasite eggs can influence a parasite's choice of nest in which to lay eggs (Honza et al. 2014), we visually modeled the avian perceived difference in color. Following the methods of (Cassey et al. 2009), we took reflectance spectra measurements from the intact eggshells used for volume analyses with an Ocean Optics USB2000 Miniature Fiber Optics Spectrometer (Dunedin, FL, USA). We recorded eggshell reflectance from three locations (the equatorial region and each pole) and calculated a mean reflectance spectrum for each egg from these three measurements (Stoddard and Stevens 2011).

We tested whether females laid their first physiologically committed egg into a nest containing zebra finch eggs, rather than Bengalese finch eggs, more frequently than predicted by chance (50%) with a two-tailed binomial distribution. To calculate whether zebra finches perceived a visual difference between the two egg types, we used the Vorobyev–Osorio model for tetrachromatic ultraviolet sensitive visual perception (Vorobyev and Osorio 1998), with a closed nest irradiance spectrum to define the ambient light environment (extracted from Cassey et al. 2009), in Avicol v2 (Gomez 2010). This model provides the “just noticeable difference” (JND) between the two egg types; JNDs >1 can be visually distinguished by the zebra finch (Cassey et al. 2009).

## Results

Seven females laid their physiologically committed egg in the nest containing zebra finch eggs and ten laid in the nest containing Bengalese finch eggs (Fig.2B). This rate of parasitism of zebra finch nests (41%) did not differ from the binomial expectation of 50% (*P * = * * 0.63).

The Bengalese finch eggs' mean volume (1.37 cm^3^ ± 0.02 SE) was larger than the zebra finch eggs' mean volume (1.13 cm^3^ ± 0.05 SE; two sample, unequal variance *t*_11_ = 4.37, *P * = * * 0.001). In the perceptual modeling, neither the mean achromatic JND value nor the mean chromatic JND value was different from 1 (achromatic JND: 1.19 ± 0.19 SE, *t*_9_ = 0.996, *P * = * * 0.35; chromatic JND: 0.78 ± 0.14 SE, *t*_9_ = 1.638, *P * = * * 0.14). For the achromatic contrast matrix, 49% (*n*  = 100) of zebra finch versus Bengalese finch comparisons and 36% (*n*  = 45) of zebra finch versus zebra finch comparisons had JND >1 (*χ* ^2^ = 1.76, *P*  > 0.1). However, for the chromatic contrast matrix, JND was >1 in 23% of zebra finch versus Bengalese finch comparisons and 4% of zebra finch versus zebra finch comparisons (*χ* ^2^ = 16.51, *P*  < 0.0001), implying a potentially perceivable difference between a significant subset of con- and heterospecific eggs.

## Discussion

Following experimental nest destruction, zebra finches showed no preference for laying a physiologically committed egg in a nest containing zebra finch eggs versus a nest containing Bengalese finch eggs. These results provide direct support for the Hamilton–Orians hypothesis in that nest destruction can cause facultative brood parasitism of both intraspecific (Feare 1991; McRae 1998; Shaw and Hauber 2009, 2012) and interspecific nests (this study). Thus nest destruction could be an evolutionarily viable pathway toward obligate interspecific brood parasitism in birds.

Some brood parasites show a preference for parasitizing nests that contain eggs with similar phenotypes to their own (Cherry et al. 2007; Honza et al. 2014); alternatively, facultative brood parasites may prefer nests with more desirable egg characteristics (e.g., brighter or larger: Tinbergen 1951). Zebra finches in this study laid eggs randomly in conspecific versus heterospecific nests, even though heterospecific eggs were distinguishable from conspecific eggs in traits including size and, for some eggs, coloration. It is possible that zebra finches may discriminate when more cues are available to differentiate nests, such as nest structures that differ to their own, or the presence of con- or heterospecific nest owners. In this study, all nest structures were identical (enclosed nests lined with hay and wool), which may be biologically plausible if nest predation resulted in preferential or indiscriminate brood parasitism of a conspecific or a closely related heterospecific with similar nest architecture. Additionally, active defense by nest owners is likely to interfere with nest inspection and successful laying of a physiologically committed egg (Shaw and Hauber 2012). Thus, for females faced with an immediate need for a host nest in which to lay a physiologically committed egg, host presence may indicate nest suitability; however, opportunities for entering the nest and inspecting its contents will likely be limited. Nonetheless, future studies are needed to elucidate whether nonrandom laying persists when more cues (e.g., the presence of con- and heterospecific hosts) are available for nest discrimination.

A previous attempt to demonstrate that nest destruction can lead to facultative intraspecific brood parasitism in the wild was unsuccessful (Rothstein 1993), and it was suggested that this may have been because the studied species very rarely brood parasitizes conspecifics (Yezerinac and Dufour 1994). By contrast, intraspecific parasitism is common in zebra finches in both captive (Schielzeth and Bolund 2010) and wild (Birkhead et al. 1990; Griffith et al. 2010) populations. Thus, our results support the hypothesis that facultative intraspecific brood parasitism is a predisposing attribute to obligate interspecific brood parasitism. This hypothesis is further supported by other facultative avian interspecific brood parasites behaving as intraspecific parasites, as well as the pattern that facultative intraspecific brood parasitism occurs in the same avian families as obligate interspecific brood parasites (Lyon and Eadie 1991; Yom-Tov 2001). In contrast, a comparative analysis did not find support for this hypothesis in altricial species (Yom-Tov and Geffen 2006), as the ancestral state for the majority of lineages containing obligate interspecific brood parasites was most likely to be a nonparasitic, normally breeding mode. However, such disagreements between experiments and comparative data will likely endure until a more comprehensive understanding of the breeding ecologies (e.g., a more complete dataset on the evolutionary prevalence of facultative intraspecific brood parasitism) of the relevant species is achieved (Feeney et al. 2014).

Although facultative intraspecific parasitism is common in wild zebra finches (Birkhead et al. 1990), our study suggests that there may not have been selection for the ability to use nest contents to discriminate suitable host nests in zebra finches. In the absence of this discrimination ability, nest predation, competition, and other sources of nest loss may promote not only intraspecific brood parasitism, but may also provide a direct pathway for the evolution of interspecific brood parasitism.
